# Breast conservation and oncoplastic surgery are associated with improved quality of life

**DOI:** 10.3389/fonc.2024.1465769

**Published:** 2024-10-18

**Authors:** Daniel Barbalho, Natalia Polidorio, Lincon Mori, Alfredo Barros, Marcelo Sampaio, Sandro Melo, Amilcar Assis, Pamela Bioni, Giovanna Miziara, Murilo Fraga, Felipe Andrade

**Affiliations:** ^1^ Department of Breast Surgical Oncology, Hospital Sírio-Libanês, Brasília, Brazil; ^2^ Department of Breast Surgical Oncology, Hospital Sírio-Libanês, São Paulo, Brazil; ^3^ Department of Plastic and Reconstructive Surgery, Hospital Sírio-Libanês, São Paulo, Brazil

**Keywords:** breast neoplasms, surgical oncology, quality of life, psychological well-being, oncoplastic breast conservation surgery

## Abstract

**Introduction:**

Local treatment can be distressful to breast cancer patients. We aimed to evaluate how different types of local treatment impact the quality of life of patients.

**Methods:**

In this retrospective cohort study, one-year postoperative Breast-Q Satisfaction with Breasts scores were used as a surrogate for Quality of Life. Linear regression was used to estimate the impact of breast conservation, oncoplastic surgery, breast reconstruction, and radiation therapy on Breast-Q scores. All analyses were adjusted for multiple covariates.

**Results:**

Of the 711 eligible patients, 349 female patients answered both the pre- and one-year postoperative questionnaires and were included in the final analysis. In total, 237 (68%) patients underwent breast-conserving surgeries and 112 (32%) underwent mastectomies. All mastectomy patients underwent breast reconstruction and 176 (74% of breast-conserving surgeries) underwent concomitant oncoplastic surgery. After multivariate analysis, mastectomy was associated with lower scores compared to breast-conserving surgery (-21.3; 95%CI: -36.2, -6.4, p=0.005), and oncoplastic surgery was associated with higher scores (9.2; 95%CI: 0.8, 17.6, p=0.032). There was a tendency for higher scores with the use of flaps in breast reconstruction and a tendency for lower scores with the use of radiation therapy, but the difference was not significant.

**Conclusions:**

Breast-conserving surgery is associated with better quality of life than mastectomy. Additionally, oncoplastic surgery is associated with a better quality of life than standard breast-conserving surgery. Patients should be counseled whenever multiple options for surgery are possible, and efforts should be made to increase the availability of trained surgeons in oncoplastic techniques.

## Introduction

Breast Cancer affects millions of women worldwide and local therapy is an integral part of its treatment. Since the era of Halstedian mastectomy without reconstruction to the current era of breast conservation and oncoplastic surgery (1), a lot of progress has been made to mitigate the harm on self-esteem and social confidence of these women.

However, after the increasing availability of germline testing and the observation that a small subset of women with pathogenic variants in high penetrance genes benefit from bilateral mastectomies (2–5), we saw an increase in the indication for mastectomies even in situations where the benefit of this procedure is unclear (6).

This study aimed to investigate the real impact of different types of local therapy on the quality of life of patients with breast cancer using one-year postoperative Breast-Q Satisfaction with Breasts scores as a surrogate measure.

## Materials and methods

This was a retrospective cohort study of a prospectively collected database, following International Consortium for Health Outcomes Measurement (ICHOM) protocols. ICHOM was founded in 2012 by Harvard Business School, the Boston Consulting Group, and the Karolinska Institute as an international effort to assess the quality of healthcare using patient-centered values (7).

The purpose of this study was to assess the impact of different types of surgery on the quality of life of Breast Cancer patients using Breast-Q Satisfaction with Breasts scores obtained one year after the first oncological surgery as a surrogate measure of quality of life. The Breast-Q is a validated questionnaire specifically developed to assess surgical outcomes in patients with Breast Cancer that encompasses psychological, social, and physical aspects reported by patients (8).

The inclusion criteria were Breast Cancer patients aged 18 years or older who received surgical treatment from December 2017 to December 2021 and spontaneously agreed to be followed by ICHOM protocols. The exclusion criteria were cognitive barriers to answering the Breast-Q questionnaires, missing Breast-Q scores, and a second oncological breast surgery in less than a year of the index surgery.

Demographic, pathological, and surgical variables collected included age, education, marital status, body mass index, diabetes, histology, tumor size, number of positive lymph nodes, tumor subtype, presence of bilateral cancer, use of chemotherapy, radiation therapy, hormone therapy, development of postoperative complications, and preoperative breast-Q satisfaction with breast scores. Only major complications, defined as those that required readmission, were included. The patients were divided into two groups: Breast Conservation and Mastectomy. The type of axillary surgery, oncoplastic surgery, and mastectomy reconstruction were also recorded.

Oncoplastic surgery was defined according to the consensus of the American Society of Breast Surgeons: a breast conservation surgery incorporating an oncologic partial mastectomy with ipsilateral defect repair using volume displacement or volume replacement techniques with contralateral symmetry surgery as appropriate. In this study, local tissue rearrangement techniques or level 1 oncoplastic surgery was classified as No Oncoplastic Surgery. Local Flaps included pedicled reduction mammaplasty designs or level 2 oncoplastic surgery. Volume replacement techniques were divided into implants, implants with flaps or flaps alone accordingly (9).

Continuous data are presented as median and interquartile range. Categorical data were presented as percentiles. Univariate analysis was performed using the Wilcoxon rank-sum test or Fisher’s exact test for continuous and categorical data. For the purpose of multivariable analysis, linear regression was performed adjusting for the preoperative Breast-Q Satisfaction with Breasts scores and every variable that was significantly different between the groups. Additional variables were included only if they improved the performance of the model according to the adjusted R-squared value. The variables included in the final model were age, education, marital status, body mass index, T stage, N stage, tumor subtype, bilateral cancer, radiation therapy, axillary surgery, complications, and preoperative Breast-Q scores. Missingness was not related to the postoperative scores. Hence, it was assumed to be missing at random and a missing indicator category was used. All analyses were performed using R version 4.3.2.

There was no coercion for patients who followed the ICHOM protocols. All patients agreed to disclose their data for quality control and research purposes. The Institutional Review Board approved data collection, as no new medical intervention would be pursued, and confidentiality would be preserved. Data were de-identified for statistical analysis and protected from reidentification.

## Results

**Figure 1:**
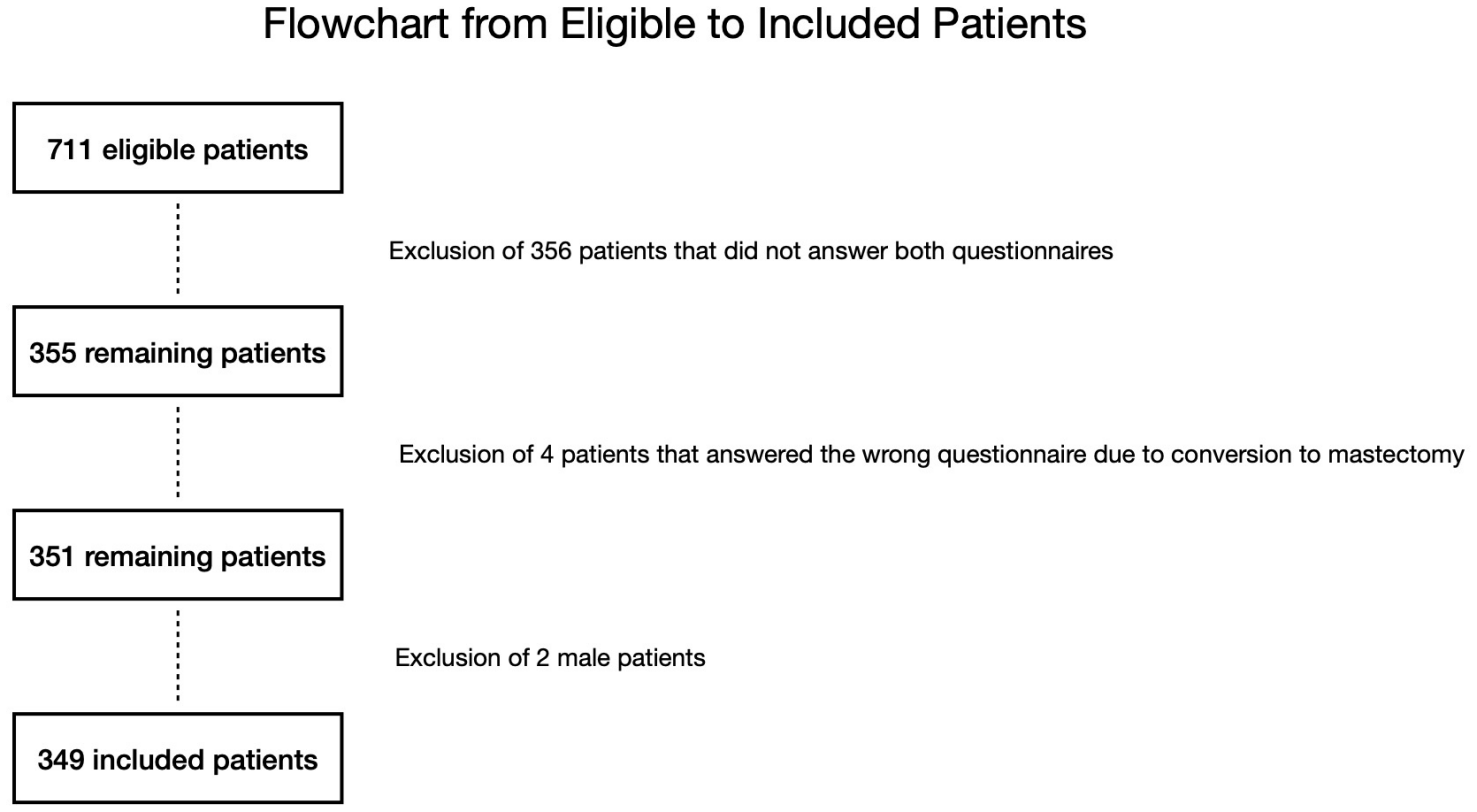
Flowchart from Eligible to Included Patients.

From December 2017 to December 2022, there were 711 eligible patients. Of these, 674 answered the preoperative Breast-Q questionnaire and 477 answered it one-year postoperatively. Of these, 355 answered both the pre- and postoperative questionnaires. Two male patients and four patients who answered the wrong questionnaire due to conversion to mastectomy were excluded from the study. Finally, 349 patients were included in the final analysis (**Figure 1**).

As shown in **Table 1**, the median age of the entire sample was 54 years, and mastectomy patients were significantly younger. The majority of participants had at least a college education (83%) and were married (77%). Only 7% of the patients had diabetes, and the median body mass index was 25 kg/m^2^. Both groups had a median preoperative Breast-Q score of 100 points. There were no differences according to the tumor histology or subtype. However, mastectomy patients had significantly larger tumors and a higher axillary burden. Only 2% of patients had bilateral synchronous cancers. We know that 21% had received chemotherapy at our institution, but the remaining patients could have received chemotherapy outside our institution as expected, if indicated. Eighty-five percent of patients received hormonal therapy according to tumor subtype, and 40% of mastectomy patients received post-mastectomy radiation therapy. All mastectomy patients underwent immediate breast reconstruction, and 60% of these patients received implants only. Seventy-four percent of breast conservation patients received oncoplastic surgery, and the majority (69%) received local flaps only. Mastectomy patients underwent significantly more axillary dissection due to a higher disease burden (23% of mastectomy patients), and 10% of the entire sample received no axillary intervention due to other histology such as sarcomas or large Phyllodes tumors. We observed 3% major complications in the mastectomy group. The only major complication observed in the breast-conserving group was deep vein thrombosis. We had more than 10% missing data on chemotherapy and tumor subtype. As stated previously, some patients may have received chemotherapy elsewhere. As for tumor subtype, some had a histology that did not warrant immunohistochemistry, and some had a pathology assessment outside our institution. Nonetheless, missingness was not related to the outcome and a missing indicator category was used. (**Table 1**)

**Table 1:** Demographics, Pathological, and Surgical Features.

		Breast-Conserving Surgery	Mastectomy	*p*
**n**		237	112	
Age (median in years [IQR^a^])		57.80 [48.30, 66.40]	50.20 [41.75, 60.05]	<0.001
Education (%)	Pre-College	29 (12.2)	15 (13.4)	0.854
	College	173 (73.0)	78 (69.6)	
	Graduate	26 (11.0)	13 (11.6)	
	Missing	9 (3.8)	6 (5.4)	
Marital Status (%)	Single	24 (10.1)	11 (9.8)	0.850
	Married	183 (77.2)	85 (75.9)	
	Divorced	15 (6.3)	10 (8.9)	
	Widow	12 (5.1)	4 (3.6)	
	Missing	3 (1.3)	2 (1.8)	
Body-Mass Index (median in kg/m2 [IQR^a^])		25.04 [22.83, 27.92]	24.36 [22.29, 27.60]	0.111
Diabetes (%)	No	222 (93.7)	103 (92.0)	0.651
	Yes	15 (6.3)	9 (8.0)	
Pre-operative Breast-Q Score (median [IQR^a^])		100.00 [82.00, 100.00]	100.00 [71.00, 100.00]	0.830
Histology (%)	DCIS^b^	43 (18.1)	25 (22.3)	0.866
	No Special Type	145 (61.2)	68 (60.7)	
	Lobular	20 (8.4)	8 (7.1)	
	Other	27 (11.4)	10 (8.9)	
	Missing	2 (0.8)	1 (0.9)	
Tumor Size (median in mm [IQR^a^])		12.00 [8.00, 18.00]	22.00 [10.25, 30.00]	<0.001
Pathological T (%)	T0	57 (24.1)	27 (24.1)	<0.001
	T1	136 (57.4)	35 (31.2)	
	T2	27 (11.4)	40 (35.7)	
	T3	2 (0.8)	4 (3.6)	
	Missing	15 (6.3)	6 (5.4)	
Pathological N (%)	N0	191 (80.6)	65 (58.0)	<0.001
	N1	24 (10.1)	24 (21.4)	
	N2	0 (0.0)	7 (6.2)	
	N3	2 (0.8)	5 (4.5)	
	Missing	20 (8.4)	11 (9.8)	
Tumor Subtype (%)	HR^c^ Positive Her2^d^ Negative	148 (62.4)	63 (56.2)	0.783
	HR^c^ Positive Her2^d^ Positive	18 (7.6)	11 (9.8)	
	HR^c^ Negative Her2^d^ Positive	8 (3.4)	3 (2.7)	
	HR^c^ Negative Her2^d^ Negative	11 (4.6)	6 (5.4)	
	Missing	52 (21.9)	29 (25.9)	
Bilateral Cancer (%)	No	236 (99.6)	107 (95.5)	0.014
	Yes	1 (0.4)	5 (4.5)	
Chemotherapy (%)	No	0 (0.0)	0 (0.0)	0.580
	Yes	49 (20.7)	26 (23.2)	
	Missing	188 (79.3)	86 (76.8)	
Hormone Therapy (%)	No	17 (7.2)	13 (11.6)	0.379
	Yes	204 (86.1)	91 (81.2)	
	Missing	16 (6.8)	8 (7.1)	
Radiation Therapy (%)	No	0 (0.0)	67 (59.8)	<0.001
	Yes	237 (100.0)	45 (40.2)	
Axillary Staging (%)	No	33 (13.9)	3 (2.7)	<0.001
	Sentinel Node	190 (80.2)	83 (74.1)	
	Axillary Lymph Node Dissection	13 (5.5)	26 (23.2)	
	Missing	1 (0.4)	0 (0.0)	
Oncoplastic Surgery (%)	No	59 (24.9)	112 (100.0)	<0.001
	Local Flaps	121 (51.1)	0 (0.0)	
	Implant	21 (8.9)	0 (0.0)	
	Distant Flap	30 (12.7)	0 (0.0)	
	Implant with Distant Flap	4 (1.7)	0 (0.0)	
	Missing	2 (0.8)	0 (0.0)	
Mastectomy Reconstruction (%)	No	237 (100.0)	0 (0.0)	<0.001
	Implant	0 (0.0)	67 (59.8)	
	Implant with Flap	0 (0.0)	23 (20.5)	
	Flap	0 (0.0)	22 (19.6)	
Complications (%)	No	236 (99.6)	101 (90.2)	<0.001
	Skin-Flap Necrosis	0 (0.0)	5 (4.5)	
	Implant Loss	0 (0.0)	2 (1.8)	
	Infection	0 (0.0)	1 (0.9)	
	Hematoma	0 (0.0)	2 (1.8)	
	Dehiscence	0 (0.0)	1 (0.9)	
	Deep-Vein Thrombosis	1 (0.4)	0 (0.0)	

a IQR, interquartile range.

b DCIS, ductal carcinoma in situ.

c HR, hormone receptors.

d Her2, human epidermal growth factor receptor 2.

**Figure 2:**
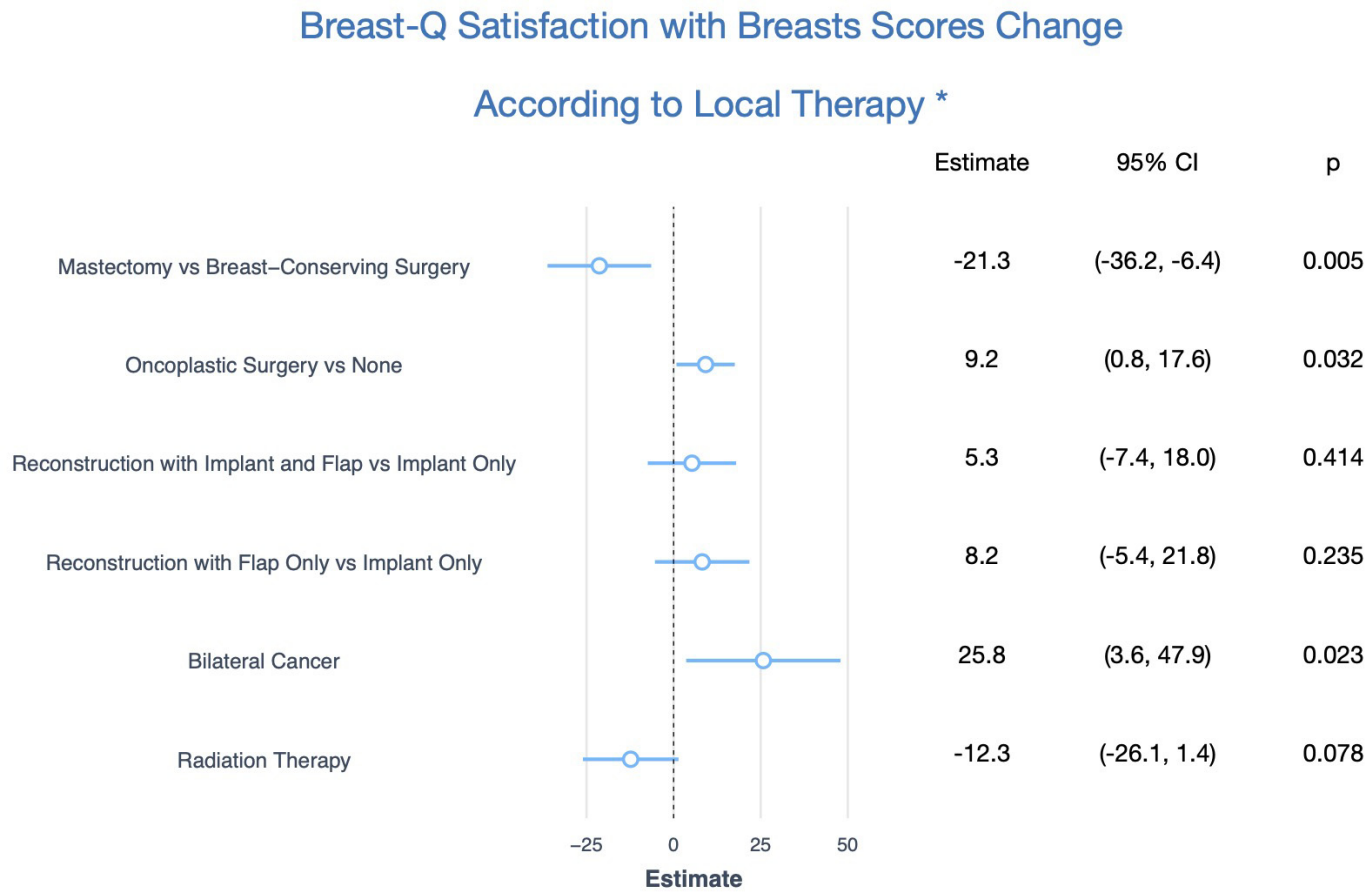
Main Findings of One-Year Postoperative Breast-Q Satisfaction with Breasts Score Change According to Local Therapy. * Adjusted for age, education, marital status, body mass index, preoperative Breast-Q score, T stage, N stage, tumor subtype, axillary surgery, and complications.

Given the observational design of our study, all results were adjusted by the preoperative Breast-Q scores, all factors that were significantly different between the groups (age, T stage, N stage, presence of bilateral cancers, axillary surgery, and complications), and factors that improved the performance of the model regardless of statistical significance (education, marital status, body mass index, and tumor subtype). The unadjusted and adjusted estimates are shown in **Table 2** and the main findings are summarized in **Figure 2**. After adjustment, mastectomy patients had significantly lower scores than breast conservation (-21.3; 95%CI: -36.2, -6.4, p=0.005). Moreover, oncoplastic surgery with local flaps was significantly associated with even higher scores than breast conservation without oncoplastic techniques (9.2; 95%CI: 0.8, 17.6, p=0.032). Regarding breast reconstruction, there was a tendency for higher scores with the use of implants and flaps compared to implants only (5.3; 95%CI: -7.4, 18.0, p=0.414), and with the use of flaps only compared to implants only (8.2; 95%CI: -5.4, 21.8, p= 0.235), but the difference was not significant. Radiation therapy was associated with lower scores (-12.3, 95%CI: -26.1, 1.4, p=0.078), albeit not significantly. Unexpectedly, bilateral cancer was significantly associated with higher scores (25.8; 95%CI: 3.6, 47.9, p=0.023). Of the six patients with bilateral cancers in our sample, five were treated with bilateral mastectomies, and one was treated with bilateral breast-conserving surgery without any oncoplastic technique (**Table 2**; **Figure 2**).

**Table 2:** Unadjusted and Adjusted Estimates of One-Year Postoperative Breast-Q Satisfaction with Breasts Score Change According to Multiple Variables.

		Unadjusted Estimate	95% CI	p	Adjusted Estimate	95% CI	p
Group	Breast Conservation	Reference			Reference		
	Mastectomy	-16.5	(-22.5, -10.5)	<0.001	-21.3	(-36.2, -6.4)	0.005
Oncoplastic Surgery	No	Reference			Reference		
	Local Flaps	16.2	(10.0, 22.4)	<0.001	9.2	(0.8, 17.6)	0.032
	Implant	2.2	(-9.8, 14.3)	0.714	-5.6	(-19.3, 8.0)	0.418
	Distant Flap	16.0	(5.7, 26.3)	0.002	8.9	(-3.0, 20.7)	0.142
	Implant with Distant Flap	26.5	(0.1, 52.9)	0.049	18.5	(-8.3, 45.3)	0.175
	Missing	26.5	(-10.6, 63.6)	0.161	21.8	(-15.1, 58.7)	0.246
Breast Reconstruction	Implant	Reference			Reference		
	Implant with Flap	4.4	(-8.2, 17.1)	0.490	5.3	(-7.4, 18.0)	0.414
	Flap	5.5	(-7.4, 18.3)	0.404	8.2	(-5.4, 21.8)	0.235
Bilateral Cancer	No	Reference			Reference		
	Yes	12.2	(-10.2, 34.5)	0.286	25.8	(3.6, 47.9)	0.023
Radiation Therapy	No	Reference			Reference		
	Yes	8.7	(1.4, 16.0)	0.020	-12.3	(-26.1, 1.4)	0.078
Pre-operative Breast-Q Score		0.1	(0.0, 0.3)	0.035	0.1	(-0.1, 0.2)	0.419
Age		0.2	(-0.1, 0.4)	0.197	0.0	(-0.2, 0.3)	0.829
Education	Pre-College	Reference			Reference		
	College	4.6	(-4.2, 13.5)	0.304	2.4	(-6.5, 11.3)	0.599
	Postgraduation	-1.0	(-12.9, 10.9)	0.871	-4.1	(-15.8, 7.7)	0.495
	Missing	-11.1	(-27.3, 5.0)	0.177	-14.7	(-31.8, 2.4)	0.091
Marital Status	Single	Reference			Reference		
	Married	0.8	(-9.0, 10.5)	0.878	0.9	(-8.6, 10.3)	0.856
	Divorced/Widow	5.1	(-7.4, 17.6)	0.424	9.2	(-3.1, 21.4)	0.143
	Missing	11.1	(-14.9, 37.1)	0.402	29.4	(1.3, 57.4)	0.040
Body-Mass Index		-0.5	(-1.2, 0.2)	0.190	-0.6	(-1.3, 0.2)	0.122
Tumor Subtype	HR^a^ Positive Her2^b^ Negative	Reference			Reference		
	HR^a^ Positive Her2^b^ Positive	0.6	(-10.2, 11.4)	0.909	2.0	(-8.4, 12.4)	0.709
	HR^a^ Negative Her2^b^ Positive	-7.7	(-24.5, 9.2)	0.372	-12.5	(-29.2, 4.2)	0.143
	HR^a^ Negative Her2^b^ Negative	6.1	(-7.6, 19.9)	0.380	8.9	(-4.4, 22.1)	0.188
	Missing	0.1	(-7.0, 7.2)	0.978	-3.8	(-11.7, 4.0)	0.340
Pathological T	T0	1.4	(-5.7, 8.5)	0.697	7.1	(-1.1, 15.3)	0.091
	T1	Reference			Reference		
	T2/T3	-13.5	(-21.0, -6.1)	<0.001	-4.0	(-12.5, 4.5)	0.359
	Missing	-0.2	(-12.5, 12.2)	0.978	-18.8	(-37.6, -0.1)	0.049
Pathological N	N0	Reference			Reference		
	N1	-7.1	(-15.5, 1.3)	0.098	2.6	(-7.8, 13.1)	0.619
	N2/N3	-22.2	(-36.9, -7.5)	0.003	1.5	(-16.5, 19.6)	0.867
	Missing	5.3	(-4.9, 15.4)	0.311	21.9	(5.9, 37.8)	0.007
Axillary Surgery	No	7.2	(-2.3, 16.7)	0.138	1.6	(-8.9, 12.0)	0.770
	Sentinel Node	Reference			Reference		
	Axillary Lymph Node Dissection	-14.4	(-23.5, -5.2)	0.002	-1.3	-11.9, 9.4)	0.816
	Missing	-3.9	(-57.5, 49.7)	0.885	-15.8	(-68.1, 36.6)	0.554
Complications	No	Reference			Reference		
	Yes	-15.7	(-31.6, 0.2)	0.053	-1.2	(-17.4, 14.9)	0.879

a HR, hormone receptors.

b Her2, human epidermal growth factor receptor 2.

## Discussion

This was an observational study evaluating the impact of local therapy on the quality of life of patients with breast cancer. Our population came from a single institution with two centers. Mastectomy patients were significantly younger than those who underwent breast-conserving surgery, although we found no evidence of more advanced disease in younger patients. We could infer that this is probably due to a higher prevalence of germline pathogenic variants in high-penetrance genes in younger patients; however, we do not have data to confirm this hypothesis. We found a higher disease burden in the mastectomy group, as expected, as well as more axillary dissection, more bilateral cancers, and more major complications among mastectomy patients. There was no difference in the preoperative Breast-Q scores between the groups.

Although not significantly, education, marital status, and body mass index improved the performance of our model. Single patients tended to have lower scores than partnered patients. College-educated patients tended to have higher scores than their less-educated peers, but postgraduation was also associated with lower scores. Satisfaction with the breast was also lower as the body mass index increased. This is in accordance with previous studies that have found the same association (10, 11).

After adjustment, mastectomy patients scored 21.3 points lower than the breast conservation group on average. This difference is clinically significant, as previous studies found the minimal clinically important difference to be 5 in the Breast-Q Satisfaction with Breasts questionnaire (12). Patients should be informed of this when multiple surgical options are available. Other studies arrived at the same conclusion as ours (13). However, when the comparison was restricted to mastectomy without reconstruction versus breast conservation with radiation, some studies have shown no significant difference in satisfaction with breasts outcome (14). In our study, 40% of the mastectomy patients received post-mastectomy radiotherapy. Radiation tended to be a predictor of worse quality of life, but this was not significant, probably due to the attenuation of the effect given that surgeons aware of the indication of radiotherapy might have chosen the use of a flap to counterbalance the detrimental effects of radiation on reconstructed breasts.

Among patients who underwent breast conservation, those who underwent oncoplastic surgery with local flaps scored 9.2 points higher than those who did not undergo oncoplastic surgery on average. This was almost twice the minimal clinically significant difference. A recent meta-analysis with more than 10,000 patients arrived at the same conclusion, with a similar magnitude of benefit (15). We do not feel that every patient undergoing breast conservation should undergo oncoplastic surgery; however, in cases where a defect is expected, all efforts should be made to achieve breast symmetry. Notably, patients who received oncoplastic breast conservation with the use of an implant tended to have lower scores. Although we did not reach statistical significance in our sample, this could be due to the deleterious effects of radiation therapy on implants.

Among the patients who underwent breast reconstruction, there was a non-significant tendency for higher scores in those who underwent flap-based reconstruction. Other studies have achieved statistical significance with a similar magnitude of benefit in this situation (10, 16). We could either be underpowered to detect such a difference, or this could be due to a diluted effect even after adjustment because patients who received a flap-based reconstruction also received more post-mastectomy radiation therapy in our sample than those who underwent implant-based reconstruction only (51% vs. 33%). We do not feel that every mastectomy patient should receive flap-based reconstruction because we did not consider donor-site morbidity associated with flaps. Implant-based reconstructions are faster, tend to have quicker recovery, and are less painful. In addition, prepectoral reconstructions can eliminate animation deformities. Nevertheless, flap scores are significantly higher and are associated with fewer complications when radiation therapy is warranted (17). Therefore, flaps should be considered in cases of postmastectomy radiotherapy.

There were only six cases of bilateral cancer in our sample. However, bilateral cancers scored 25.8 points significantly higher on average. This finding may seem counterintuitive, because it contradicts the main findings of this study. Of the six bilateral cancers, one received bilateral breast-conserving surgery without any oncoplastic technique, the remaining five received bilateral mastectomies, four received radiation therapy, and only two underwent flap-based reconstruction. This could be a false-positive finding due to the small number of patients. Nonetheless, this is not the first similar report. Two previous studies showed greater satisfaction with bilateral mastectomies than with unilateral ones (16, 18), but this result was not consistent across different studies (19). We do not wish to advocate bilateral mastectomy in all patients. However, in patients who cannot escape one mastectomy, a second mastectomy may yield better symmetry in selected patients, which could help explain some of these findings.

As this was a single-institution study, the generalizability of our results may be threatened. Another limitation of this study was its retrospective and observational design with potential residual or undetected confounders. On the other hand, all efforts were made to report our findings according to Strobe guidelines. Thorough adjustment for multiple covariates was used to minimize the risk of bias, including self-control, using the preoperative Breast-Q score of all patients.

## Conclusions

Breast conservation is significantly associated with a better quality of life, with a large clinically significant difference compared with reconstructed mastectomies, with fewer complications. Oncoplastic surgery is significantly associated with greater benefits in patients that undergo breast-conserving surgery. Patients with multiple options for surgery should be advised of this consistent benefit of breast conservation. More surgeons should be trained in oncoplastic techniques to reduce the morbidity associated with local therapy and promote welfare in patients with breast cancer.

## Data Availability

The raw data supporting the conclusions of this article will be made available by the corresponding author upon request and after institutional approval.
